# Across-rotation genetic analysis and multitrait selection in a cloned cross of *Eucalyptus urophylla* × *E*. *tereticornis*

**DOI:** 10.3389/fpls.2025.1553819

**Published:** 2025-06-03

**Authors:** Jiahong Xu, Mingming Zhou, Qijie Weng, Mei Li, Siming Gan

**Affiliations:** ^1^ State Key Laboratory of Tree Genetics and Breeding, Chinese Academy of Forestry, Beijing, China; ^2^ Key Laboratory of State Forestry Administration on Tropical Forestry Research, Research Institute of Tropical Forestry, Chinese Academy of Forestry, Guangzhou, China; ^3^ College of Forestry, Nanjing Forestry University, Nanjing, China

**Keywords:** *Eucalyptus* hybrid, across-rotation traits, genetic parameters, multi-trait genetic selection, genetic gain, selection efficiency

## Abstract

Clonal testing is crucial for estimating genetic parameters and selecting elite clones. However, few studies have tested clones over two or more rotations. In this study, a clonal trial of the sibs from a *Eucalyptus urophylla* × *Eucalyptus tereticornis* cross was used for genetic parameter analyses and multitrait selection based on traits such as across-rotation growth, wood properties, coppicing ability, and coppice chlorophyll fluorescence. Clonal repeatability (*H* ^2^) estimates for growth traits in the first rotation ranged from 0.73 (diameter at breast height at age 15) to 0.84 (single-tree volume at age 2.5) and were generally higher than those for growth traits in the second rotation (0.11–0.62). *H* ^2^ estimates for coppicing and chlorophyll fluorescence traits ranged from 0.35 to 0.57 and 0.03 to 0.53, respectively, indicating low to medium genetic control of these traits. Phenotypic correlations ( $r_{p}$ ) and additive genetic correlations ( $r_{g}$ ) were generally high among growth traits within a single rotation and among coppicing traits but varied considerably across other trait pairs. In particular, second-rotation volume at 2.5 years showed a very weak $r_{p}$ but a moderately positive $r_{g}$ with early-stage volumes from the first rotation. Multitrait selection index (SI) and multitrait genotype–idiotype distance index (MGIDI) methods were applied at a selection intensity of 15% under four scenarios, each combining two to five traits. Relative genetic gain (*RG*) and selection efficiency (*E*) estimates were positive for all traits included in multitrait selection, indicating the usefulness of all selection scenarios. Compared to SI, the MGIDI method produced slightly lower *RG* and *E* values for volumes at ages 8 and 15 years, but higher *RG* and *E* values for wood basic density and cellulose content at age 8 in the first rotation. This study has important implications for eucalypt clonal breeding and management.

## 1 Introduction

Clonal forestry, defined as the commercial deployment of identified, well-characterized forest tree clones (Burdon and Aimers-Halliday, 2006), has been successfully implemented to improve plantation productivity in many countries. In China, for example, over 60% of *Eucalyptus* L’Hérit. (family Myrtaceae Juss.) plantations are clonal (Wei, 2005), and the large-scale development of clonal forestry has increased productivity from less than 8 to over 15 m^3^ ha^−1^ year^−1^ in around 30 years (Arnold et al., 2020). Also, the genetic uniformity of clonal ramets can lead to phenotypic uniformity, which enables the managers to plan and execute clonal forestry activities much more efficiently than seedling-based plantations (Kleinschmit et al., 1993; Soares et al., 2016). Moreover, the improved productivity and phenotypic uniformity can be extended over a couple of rotations through coppice regeneration.

There can sometimes be concerns about the risks of the deployment of clones in plantation forestry, such as reduced genetic diversity, vegetative propagation failure, cultivar decline, and biotic or climatic damage. However, appropriate choice of management regimes, such as the use of an optimal number of unrelated clones (or so-called clonal composites) and effective control of possible damages, can diminish such risks (Kleinschmit et al., 1993; Stelzer and Goldfarb, 1997; Burdon and Aimers-Halliday, 2006; Rezende et al., 2019). Nowadays, clonal forestry has been implemented for a multitude of broadleaved and coniferous trees, including pure species and interspecific hybrids from the genera *Eucalyptus*, *Populus* L., *Pinus* L., and *Picea* Dietr (Wu, 2019).

Clonal testing is required for the estimation of genetic parameters and the selection of elite clones (White, 1996). Genetic parameters such as heritability (or repeatability) and trait–trait correlations are crucial for determining clonal breeding strategies and predicting genetic gains. Compared to seedling-based tests, clonal tests allow for more efficient dissection of genetic and environmental effects from general phenotypic variation due to the use of ramets of the same genotype (Foster and Shaw, 1988; Isik et al., 2003; Wu, 2019). This can improve the accuracy of breeding value prediction and elite genotype selection, thus contributing to greater genetic gains (Mullin and Park, 1992; Isik et al., 2005). In addition, simultaneous testing of clonal ramets and their ortets provides opportunities for assessing the correlation between clonal and seedling performance, which may have important implications in a breeding program (Shalizi et al., 2020). In forest trees, clone-based genetic parameters and elite selection have been reported in many species, e.g., *Eucalyptus grandis* Hill ex Maiden (Lambeth et al., 1994; Osorio et al., 2001, 2003; Amâncio et al., 2020), *Pinus taeda* L (Isik et al., 2003, 2005; Shalizi and Isik, 2019; Braga et al., 2020; Shalizi et al., 2020), and *Picea abies* (L.) H. Karst (Berlin et al., 2019; Chen et al., 2020; Zeltiņš et al., 2022). However, previous studies presented results only within a single rotation, and little is known about the corresponding information across two or more rotations.

In breeding practices, multiple traits of economic importance should be simultaneously considered for selection. Several methods exist for multitrait selection, including tandem selection, simultaneous culling, conventional index selection (Smith, 1936; Hazel, 1943), and single-environment multitrait genotype–idiotype distance index (MGIDI) selection (Olivoto and Nardino, 2021). Of these, conventional index selection involves the construction of a selection index that is a linear combination of multiple traits and their relative weights. This method has a long history of application to tree breeding (Cotterill and Dean, 1990) and is still the method of choice for selection in many woody species, e.g., *Eucalyptus urophylla* S. T. Blake × *E. grandis* (Bouvet et al., 2020) and *Picea glauca* (Moench) Voss (Rashidi-Jouybari et al., 2022). More recently, the MGIDI method was proposed as a novel approach to selection based on mean performances of multiple traits (Olivoto and Nardino, 2021). It has been used effectively in crops such as *Zea mays* L. (Singamsetti et al., 2023) forest trees such as *P. abies* (Alexandru et al., 2023) and *Populus simonii* Carr. × *P*. *nigra* L (Wang et al., 2024). However, its comparison to that of conventional index selection remains to be investigated.

*Eucalyptus* represents the most widely planted broadleaved tree genus in the world (there being more than 21 million ha of plantations globally; Midgley, 2013). With the development of clonal forestry, the vast majority of eucalypt planting stock is now clonal, particularly utilizing interspecific hybrid varieties. For instance, hybrid clones from *E. urophylla*, *E. grandis*, *E. tereticornis* Smith, and *E. camaldulensis* Dehnh. predominate in eucalypt plantations in China (Wei, 2005). As noted, *E. urophylla* × *E. tereticornis* clones have characteristic fast growth, high yields, and moderate resistance to typhoons and have been cultivated widely in coastal regions in southern China (Peng et al., 2013). Earlier reports have revealed significant variation in the growth and wood properties of *E. urophylla* × *E. tereticornis* clones (Gan et al., 2006; Yang et al., 2018). However, similar to those aforementioned species available for clone-based genetic parameter estimation and elite genotype selection, earlier reports are limited to one single rotation.

We hypothesized that genetic variations existed among clonal sibs even within a single cross in *Eucalyptus* and effective selection can be carried out across rotations. In the present study, a clonal trial of the sibs of an *E. urophylla* × *E. tereticornis* cross was used to conduct genetic parameter analysis and elite clone selection on such traits as across-rotation growth, wood properties of the first rotation, coppicing ability, and coppice chlorophyll fluorescence. Our objectives were to determine the clonal repeatability (*H* ^2^), trait–trait, and age–age correlations; predict the relative genetic gains (*RG*, %) from multitrait selections; and compare the selection efficiency (*E*, %) of index selection versus MGIDI method.

## 2 Materials and methods

### 2.1 Plant material and experimental design

Rooted cuttings of 403 sibs of an interspecific cross between *E. urophylla* (maternal genotype UX-30) and *E*. *tereticornis* (paternal genotype T43-05) were planted in a common garden experiment in April 2006. The experimental site was located at Gonghe Town (22°34′24″ N, 112°51′14″ E), Heshan County, Guangdong Province, China (more details as described in Yang et al., 2018). The experiment was laid out in a design of randomized complete blocks, with single-tree plots, six blocks (replicates), and a spacing of 2 m × 3 m. The last two blocks were damaged by a fire accident that occurred at the age of around five years during the first rotation, so thereafter these blocks were excluded from investigation and analyses. Moreover, 309 ortets of the sibs were planted along with the clonal experiment.

In August 2021, all trees were felled to allow for coppice regeneration of the experiment. Stumps were cut at approximately 0.1 m above ground level. Nine months after the felling, when most stumps had sprouts exceeding 2.5 m, as commonly practiced in eucalypt coppice management in China, only the highest two sprouts were retained per stump.

### 2.2 Trait measurements and calculations

Growth traits of the first rotation (prior to felling) were measured for all trees at 10, 12, and 15 years of age, including tree height (*HT*_10_, *HT*_12_, and *HT*_15_ in m, respectively) and diameter over bark at breast height (*DBH*_10_, *DBH*_12_, and *DBH*_15_ in cm, respectively). Wood basic density at age 15 (*BD*_15_, g m^−3^) was determined based on near-infrared spectroscopy predictions using a MPA spectrometer (Bruker Optik, Ettlingen, Germany), which was similar to the method of Yang et al. (2018).

Earlier growth traits and 8-year-old wood properties were investigated as per Yang et al. (2018), including tree height at ages 0.5, 1.5, 2.5, 4.5, 5.5, 6.5, and 8 years (*HT*_0.5_, *HT*_1.5_, *HT*_2.5_, *HT*_4.5_, *HT*_5.5_, *HT*_6.5_, and *HT*_8_, respectively) and diameter at breast height at 1.5, 2.5, 4.5, 5.5, 6.5, and 8 years (*DBH*_1.5_, *DBH*_2.5_, *DBH*_4.5_, *DBH*_5.5_, *DBH*_6.5_, and *DBH*_8_, respectively), as well as 8-year-old wood basic density (*BD*_8_, g m^−3^), cellulose content (*CC*_8_, %), hemicellulose content (*HC*_8_, %), Klason lignin content (*LC*_8_, %), and lignin guaiacyl-to-syringyl ratio (*G*/*S*_8_). Single-tree volumes at 1.5, 2.5, 4.5, 5.5, 6.5, 8, 10, 12, and 15 years (*V*_1.5_, *V*_2.5_, *V*_4.5_, *V*_5.5_, *V*_6.5_, *V*_8_, *V*_10_, *V*_12_, and *V*_15_ in m^3^, respectively) were calculated as *HT* *_n_* × *DBH* *_n_* ^2^/30,000 (He et al., 2012), where *HT* *_n_* (m) and *DBH* *_n_* (cm) are height and diameter at breast height of a given year *n*, respectively.

Coppicing traits were observed for each stump six months after felling, including the number of sprouts (*NS*_0.5_), height (*HTs1*_0.5_ and *HTs2*_0.5_, m), and ground diameter (*GDs1*_0.5_ and *GDs2*_0.5_, cm) of the two highest sprouts, as well as coppicing potential (*CP*_0.5_). Specifically, coppicing potential was evaluated by combining the number and the growth condition of sprouts, and *CP*_0.5_ was scored using a six-grade scale based on the classification of *NS*_0.5_ values: grades 1, 2, 3, 4, 5, and 6 for 1–4, 5–8, 9–12, 13–16, 17–20, and ≥ 21 sprouts, respectively, with modification by increase or decrease of one grade in respect of the sprout growth condition.

Chlorophyll fluorescence and content measurements were performed in triplicate for three fully expanded young leaves per stump in August 2022, when the earliest sprouts were about one year old. A portable chlorophyll fluorometer PAM-2500 (Heinz Walz GmbH, Effeltrich, Germany) was used to measure chlorophyll fluorescence traits. Each leaf was dark-adapted for 30 min before determining the initial (*F*_0_), maximal (*F*_m_), and variable (*F*_v_ = *F*_m_ – *F*_0_) fluorescence, as well as the maximal quantum yield of photosystem II [*Y(II)* = *F*_v_/*F*_m_]. After light-adapted conditions (≥ 800 µmol photons m^−2^ s^−1^) for at least 25 min, steady-light chlorophyll fluorescence (*F*_s_), maximal fluorescence (*F*_m_′), variable fluorescence (*F*_v_′ = *F*_m_′ – *F*_s_), maximal quantum yield of photosystem II [*Y(II)* *′* = *F*_v_′/*F*_m_′], and photosynthetic electron transport rate (*ETR*) were recorded also using PAM-2500. Nonphotochemical quenching (*NPQ*) was computed as *F*_m_/*F*_m_′ – 1. In addition, a hand-held chlorophyll meter SPAD-502 (Spectrum Technologies Inc., Plainfield, IL, USA) was used to detect leaf chlorophyll content as SPAD reading (*SPADR*).

Growth traits of the two sprouts retained per stump were measured when the coppice age was around 1.5 and 2.5 years. For the age of 1.5 years, the higher and lower sprouts per stump were measured for height (*HTs1*_1.5_ and *HTs2*_1.5_ in m, respectively) and diameter at breast height (*DBHs1*_1.5_ and *DBHs2*_1.5_ in cm, respectively). The individual volume of each of the two sprouts (*Vs1*_1.5_ and *Vs2*_1.5_, m^3^) was calculated similarly as mentioned above, and *Vs1*_1.5_ and *Vs2*_1.5_ were added up to represent the single-ramet volume (*Vs*_1.5_, m^3^). Moreover, branch angle (*BAs*_1.5_, °) relative to the stem was determined by averaging those of the four branches, each being the biggest in the up, down, left, or right direction of a row. The height of the lowest live branch (*HTLBs*_1.5_, m) was observed across the two sprouts of a ramet, and number of branches (*NBs*_1.5_) was counted only for the higher sprout. Crown width was measured along row and column directions (*CWs1*_1.5_ and *CWs2*_1.5_, m), which were subsequently used for calculating crown projected area (*CPAs*_1.5_ = *CWs1*_1.5_ × *CWs2*_1.5_ × π/4, m^2^) assuming an elliptical shape. Crown length (*CLs*_1.5_, m) or depth was estimated as the difference between the height of the higher sprout (*HTs1*_1.5_ or *HTs2*_1.5_) and *HTLBs*_1.5_. For the age of 2.5 years, sprout height (*HTs1*_2.5_ and *HTs2*_2.5_, m), diameter at breast height (*DBHs1*_2.5_ and *DBHs2*_2.5_, cm), and volume (*Vs1*_2.5_ and *Vs2*_2.5_, m^3^) as well as single-ramet volume (*Vs*_2.5_, m^3^) were measured or calculated similarly as conducted above for 1.5 years of age. In a few cases, only one sprout remained per stump and was consequently measured.

### 2.3 Statistical analyses

Statistical analyses were performed using RStudio (RStudio Team, 2021) in R version 4.1.3 (R Core Team, 2022). Trait means, along with their standard deviations (SD) and coefficients of variation (CV), were calculated. In addition, *t*-tests were conducted to evaluate the significance of differences between ramet means and ortet values.

#### 2.3.1 Variance components and clonal repeatability

For each of the traits, variance components were estimated using the *lmer()* function of the lme4 statistical package (Bates et al., 2015) following the linear mixed model:


$$y_{ij}=\mu +G_{i}+B_{j}+E_{ij}$$

where *Y_ij_* is the trait value of the *i*th genotype (clonal sib) in the *j*th block, *μ* represents the overall mean, *G_i_* is the additive genetic effect (random) of the *i*th genotype, *B_j_* is the effect (fixed) of the *j*th block, and *E_ij_* is the residual error. The genotypic variance component ( $\sigma_{g}^{\;2}$ ) and residual error variance component ( $\sigma_{e}^{\;2}$ ) were used to calculate the coefficient of genetic variation (CV_g_) as $(\delta_{g}/\mu )\times 100\%$ and *H* ^2^ as $\sigma_{g}^{\;2}/\left(\sigma_{g}^{\;2}+\sigma_{e}^{\;2}/r\right)$ , where *μ* and *r* represents the overall mean and the number of blocks, respectively.

#### 2.3.2 Phenotypic and additive genetic correlations

Phenotypic correlations ( $r_{p}$ ) and additive genetic correlations ( $r_{g}$ ) between trait pairs were estimated as:


$$r_{p}=\sigma_{p_{x}p_{y}}/\sqrt{\sigma_{p_{x}}^{\;2}\sigma_{p_{y}}^{\;2}}$$

$$r_{g}=\sigma_{g_{x}g_{y}}/\sqrt{\sigma_{g_{x}}^{\;2}\sigma_{g_{y}}^{\;2}}$$

where $\sigma_{{\text{p}}_{\text{x}}{\text{p}}_{\text{y}}}$ and $\sigma_{g_{x}g_{y}}$ are the estimated phenotypic and genotypic covariances between traits *x* and *y*, $\sigma_{p_{x}}^{\;2}$ and $\sigma_{p_{y}}^{\;2}$ represent phenotypic variance components of traits *x* and *y*, and $\sigma_{g_{x}}^{\;2}$ and $\sigma_{g_{y}}^{\;2}$ represent genotypic variance components of traits *x* and *y*, respectively. Genotypic and phenotypic covariances were computed using *asreml()* function, and $r_{p}$ , $r_{g}$ , and their standard error (SE) were then calculated using *vpredict()* function in ASReml (Butler et al., 2017).

#### 2.3.3 Multitrait index selection and multitrait genotype–idiotype distance index selection

Multitrait selections were conducted for ages 8 and 15 years of the first rotation, combining the two economic traits *CP*_0.5_ and *Vs*_2.5_ of the second rotation. In China, age 8 is the approximate rotation length of eucalypt plantations established for veneer and pulp industries, while age 15 is one of the rotation periods recommended for large-size eucalypt timber production (Chen et al., 2017). In respect of the traits of economic importance, *V*_8_, *BD*_8_, and *CC*_8_ for age 8 and *V*_15_ and *BD*_15_ for age 15 were employed for the across-rotation multitrait selection. Also, these traits were subject to additional single-rotation multitrait selection. Moreover, single-trait selection for *V*_1.5_, *V*_2.5_, *V*_4.5_, *V*_5.5_, *V*_6.5_, *V*_8_, *BD*_8_, *CC*_8_, *V*_10_, *V*_12_, *V*_15_, *DB*_15_, *CP*_0.5_, *Vs*_1.5_, and *Vs*_2.5_ was performed based on the best linear unbiased prediction (BLUP) of clonal breeding values (*BV*). For all the selections, a selection intensity of 15% was adopted.

Multitrait selection index (SI) was calculated following the method of Smith (1936) and Hazel (1943):


$$SI=\sum_{i=1}^{n}b_{i}x_{i}$$

where $x_{i}$ is clonal phenotypic mean values of trait *i* and $b_{i}$ is index coefficient for trait *i* derived from:


$$b=P^{-1}Gw$$

where P and G are the phenotypic and genetic variance–covariance matrices, respectively, and w is the vector of relative economic weights each defined for an objective trait using an equal-emphasis approach (Cotterill and Dean, 1990):


$$w_{i}=1/\sigma_{pi}$$

where $\sigma_{pi}$ is the phenotypic standard deviation of trait *i*.

For MGIDI selection, the MGIDI value of each clone was estimated for the objective traits based on BLUP (Olivoto and Nardino, 2021). Four main steps were carried out (Olivoto and Nardino, 2021), namely, rescaling the objective traits each into a 0–100 range, estimating the factorial score of each clone to group correlated traits into factors, planning the ideotype (clone) that has the highest rescaled value for each trait, and calculating the MGIDI of clone *i* (MGIDI_i_) as:


$$MGIDI_{i}={\left[\sum_{j=1}^{f}{\left(\gamma_{ij}-\gamma_{j}\right)}^{2}\right]}^{0.5}$$

where $\gamma_{ij}$ is the score of clone *i* in factor *j*, and $\gamma_{j}$ is the score of factor *j* of the ideotype. The lower the MGIDI of a clone, the closer the clone is to the ideotype. The function *mgidi()* of metan 1.18.0 (Olivoto and Lúcio, 2020) was used for MGIDI calculation.

#### 2.3.4 Relative genetic gain and selection efficiency

*RG* for a specific trait was calculated according to White et al. (2007):


$$RG\;\left(\%\right)=\frac{H^{2}\left(M_{s}-M_{o}\right)}{M_{o}}\times 100$$

where *H* ^2^ is clonal repeatability, $M_{s}$ is the phenotypic mean of selected clones, and $M_{o}$ is the overall phenotypic mean of all the clones.

*E* of an earlier selection (usually on a single trait) relative to a later selection (usually on multiple traits) was expressed as:


$$E\;\left(\%\right)=\frac{H^{2}\left(M_{s1}-M_{o}\right)}{M_{o}}÷\frac{H^{2}\left(M_{s2}-M_{o}\right)}{M_{o}}\times 100=\frac{M_{s1}-M_{o}}{M_{s2}-M_{o}}\times 100$$

where *H* ^2^ is clonal repeatability of a trait in later selection, $M_{s1}$ and $M_{s2}$ are the later phenotypic means of earlier and later selected clones, respectively, and $M_{o}$ is the later overall phenotypic mean of all the clones. Similarly, efficiency between selections at the same age was calculated.

## 3 Results and discussion

### 3.1 Trait variation

There were 318 (78.9%), 292 (72.5%), and 275 (67.0%) clones that survived at age 8, age 15 of the first rotation, and age 2.5 of the second rotation, respectively. Of the 54 traits investigated, *BD*_15_ and all the 15 growth traits of the first rotation, all the six coppicing traits, and nine of the 18 second-rotation growth traits (exclusive of *HTs2*_1.5_, *DBHs2*_1.5_, *Vs2*_1.5_, *BAs*_1.5_, *NBs*_1.5_, *HTs1*_2.5_, *HTs2*_2.5_, and *DBHs1*_2.5_) showed significant differences (*p* ≤ 0.001, 0.01, or 0.05) among clones. These results indicate high progeny phenotypic variability for these traits, while most sprout leaf chlorophyll-related traits (7/11 with *F*_0_, *F*_s_, *NPQ*, and *SPADR* excluded) proved to be not significant (**Supplementary Table 1**). This corroborates our earlier finding of significant clonal difference within the same cross for earlier growth (tree height and diameter at breast height across 0.5–8 years) and 8-year-old wood chemical properties (Yang et al., 2018). Similarly, significant differences among clonal sibs within a single cross were noticed for growth and leaf traits in *Populus deltoides* Bartr. ex Marsh. × *P. nigra* (Marron and Ceulemans, 2006) and (*Populus pseudo-simonii* Kitag. × *P*. *nigra*) × *Populus beijingensis* W. Y. Hsu (Liao et al., 2016) for branching in *P. taeda* (Xiong et al., 2014), but not for leaf chlorophyll traits such as *F*_v_/*F*_m_ [*Y*(*II*)] in (*P. pseudo-simonii* × *P*. *nigra*) × *P*. *beijingensis* (Liao et al., 2016).

Meanwhile, growth traits of both rotations presented generally larger coefficients of variation, ranging from 6.9% in *BAs*_1.5_ to 57.2% in *Vs1*_1.5_, as compared to those of leaf chlorophyll traits and *BD*_15_ (between 2.6% in *Y(II)* and 27.7% in *F*_v_′ except the extremely high case of 121.1% in *NPQ*; **Supplementary Table 1**). For most traits, high phenotypic variability together with considerable coefficients of variation may suggest good potential for clone selection for future deployment programs.

Significant differences (*p* ≤ 0.001, 0.01, or 0.05) between ramet means and ortet values were found for most traits, including nine, six, three, and 10 of the 15 first-rotation growth, six coppicing, 11 chlorophyll, and 21 second-rotation growth traits, respectively (**Supplementary Table 2**). For the 28 traits with significant differences, ortets outperformed their ramet means in 17 traits, including eight, six, and three of the first-rotation growth, coppicing, and second-rotation growth traits, respectively (**Supplementary Table 2**). Several previous studies reported better growth of seedlings than rooted cutting progeny originating from the same full-sib families in *Eucalyptus* (Sasse and Sands, 1997; Costa e Silva et al., 2013; van den Berg et al., 2015) and *Pinus* (Antony et al., 2014; Quesada et al., 2017; Shalizi et al., 2020). These studies attributed the growth difference to the poor root structure of rooted cuttings, indicating the presence of propagation effects specific to some clones (C-effects; Borralho and Kanowski, 1995). Even so, significantly higher or nonsignificantly different performance of ramets as compared to seedlings was shown in the current study in a couple of the growth traits (e.g., those of ages 10 and 12 of the first rotation, and age 2.5 of the second rotation, except only *HTs1*_2.5_; **Supplementary Table 2**). In forest trees, occasional cases of nonsignificantly different or better clonal performance were also reported for certain growth traits in *E. grandis* × *E. urophylla* hybrid (Sasse and Sands, 1997) and *P. taeda* full-sib progeny (Quesada et al., 2017).

### 3.2 Variance components and repeatability estimates

Estimates of *σ*_g_^2^ were significant (*p* ≤ 0.001, 0.01, or 0.05) for all the traits of the first rotation, coppicing traits, 11 of the 21 second-rotation growth traits, and three of the 11 chlorophyll traits (**Table 1**), which was almost coincident with the ANOVA results (**Supplementary Table 1**). σ_g_^2^ varied largely with the trait, e.g., CV_g_ being 2.4%, 9.4%–44.5%, 10.5%–20.0%, 0.9%–16.5%, and 1.3%–23.2% for *BD*_15_, the first-rotation growth, coppicing, chlorophyll, and the second-rotation growth traits, respectively (**Table 1**).

**Table 1:** Clonal additive genetic variance components ( $\sigma_{g}^{\;2}$ ), their standard errors (SE), coefficients of variation (CV_g_, %), and repeatabilities (*H* ^2^ ± SE) for 54 traits investigated in a cloned *E*. *urophylla* × *E*. *tereticornis* cross.

Trait	$\sigma_{g}^{\;2}$ (± SE)	CV_g_ (%)	*H* ^2^ (± SE)
Growth of the first rotation			
*V*_1.5_ (m^3^)	3.86 (± 0.39) × 10^−5***^	31.5	0.82 (± 0.03)
*V*_2.5_ (m^3^)	1.38 (± 0.13) × 10^−4***^	34.7	0.84 (± 0.04)
*V*_4.5_ (m^3^)	1.38 (± 0.15) × 10^−3***^	42.7	0.82 (± 0.03)
*V*_5.5_ (m^3^)	2.65 (± 0.29) × 10^−3***^	44.5	0.81 (± 0.03)
*V*_6.5_ (m^3^)	3.41 (± 0.47) × 10^−3***^	37.4	0.77 (± 0.03)
*V*_8_ (m^3^)	0.01 (± 0.68 × 10^−3^)^***^	39.2	0.79 (± 0.03)
*HT*_10_ (m)	4.54 (± 0.68)^***^	9.4	0.77 (± 0.03)
*DBH*_10_ (cm)	10.18 (± 1.37)^***^	18.2	0.78 (± 0.03)
*V*_10_ (m^3^)	0.01 (± 0.16 × 10^−2^)^***^	36.5	0.75 (± 0.03)
*HT*_12_ (m)	4.96 (± 0.75)^***^	9.7	0.78 (± 0.04)
*DBH*_12_ (cm)	11.39 (± 1.56)^***^	18.6	0.78 (± 0.03)
*V*_12_ (m^3^)	0.01 (± 0.20 × 10^−2^)^***^	37.0	0.74 (± 0.03)
*HT*_15_ (m)	7.42 (± 1.21)^***^	11.1	0.81 (± 0.04)
*DBH*_15_ (cm)	9.67 (± 1.54)^***^	16.2	0.73 (± 0.03)
*V*_15_ (m^3^)	0.02 (± 0.33 × 10^−2^)^***^	36.1	0.74 (± 0.04)
Wood density of the first rotation			
*BD*_15_ (g/cm^3^)	1.90 (± 0.61) × 10^−4***^	2.4	0.65 (± 0.03)
Coppicing			
*NS*_0.5_	7.20 (± 1.84)^***^	20.0	0.54 (± 0.02)
*HTs1*_0.5_ (m)	0.06 (± 0.02)^***^	11.0	0.47 (± 0.01)
*HTs2*_0.5_ (m)	0.08 (± 0.02)^***^	13.3	0.57 (± 0.02)
*GDs1*_0.5_ (cm)	4.48 (± 2.07) × 10^−4*^	10.5	0.35 (± 0.01)
*GDs2*_0.5_ (cm)	5.55 (± 1.72) × 10^−4***^	13.7	0.48 (± 0.01)
*CP*_0.5_	0.34 (± 0.09)^***^	13.1	0.54 (± 0.02)
Chlorophyll fluorescence and concentration			
*F*_0_	8.19 (± 4.88) × 10^−5^	4.1	0.30 (± 0.01)
*F*_m_	1.14 (± 6.03) × 10^−4^	1.2	0.04 (± 0.96 × 10^−4^)
*F*_v_	2.21 (± 3.66) × 10^−4^	2.2	0.12 (± 0.87 × 10^−3^)
*Y(II)*	4.49 (± 2.65) × 10^−5^	0.9	0.31 (± 0.01)
*F*_s_	5.94 (± 2.70) × 10^−4*^	6.9	0.38 (± 0.01)
*F*_m_′	1.59 (± 0.66) × 10^−3**^	7.9	0.40 (± 0.01)
*F*_v_′	7.37 (± 13.40) × 10^−5^	5.8	0.11 (± 0.72 × 10^−3^)
*Y(II)*′	1.50 (± 1.16) × 10^−4^	4.4	0.25 (± 0.37 × 10^−2^)
*ETR*	8.06 (± 51.30)	2.0	0.03 (± 0.54 × 10^−4^)
*NPQ*	0.02 (± 0.01)^*^	16.3	0.37 (± 0.01)
*SPADR*	1.68 (± 0.53)^***^	3.1	0.53 (± 0.02)
Growth of the second rotation			
*HTs1*_1.5_ (m)	0.30 (± 0.18)	7.1	0.33 (± 0.01)
*HTs2*_1.5_ (m)	0.33 (± 0.19)	7.5	0.37 (± 0.01)
*DBHs1*_1.5_ (cm)	0.43 (± 0.18)	12.0	0.41 (± 0.01)
*DBHs2*_1.5_ (cm)	0.32 (± 0.19)	10.4	0.35 (± 0.01)
*Vs1*_1.5_ (m^3^)	4.64 (± 2.39) × 10^−6*^	22.3	0.37 (± 0.01)
*Vs2*_1.5_ (m^3^)	2.95 (± 2.13) × 10^−6^	17.6	0.30 (± 0.01)
*Vs*_1.5_ (m^3^)	1.61 (± 0.66) × 10^−5**^	22.2	0.44 (± 0.01)
*BAs*_1.5_ (°)	0.67 (± 1.29)	1.3	0.11 (± 0.73 × 10^−3^)
*HTLBs*_1.5_ (m)	0.25 (± 0.06)^***^	16.3	0.62 (± 0.02)
*NBs*_1.5_	4.46 (± 3.57)	7.5	0.25 (± 0.38 × 10^−2^)
*CWs1*_1.5_ (m)	0.06 (± 0.03)^*^	8.5	0.37 (± 0.01)
*CWs2*_1.5_ (m)	0.09 (± 0.03)^**^	9.8	0.46 (± 0.01)
*CPAs*_1.5_ (m^2^)	1.51 (± 0.56)^**^	16.6	0.46 (± 0.01)
*CLs*_1.5_ (m)	0.25 (± 0.10)^**^	10.1	0.45 (± 0.01)
*HTs1*_2.5_ (m)	0.38 (± 0.41)	5.7	0.19 (± 2.23 × 10^−3^)
*HTs2*_2.5_ (m)	0.39 (± 0.45)	5.7	0.21 (± 2.81 × 10^−3^)
*DBHs1*_2.5_ (cm)	0.41 (± 0.28)	8.6	0.27 (± 4.48 × 10^−3^)
*DBHs2*_2.5_ (cm)	0.74 (± 0.33)^*^	11.7	0.44 (± 0.01)
*Vs1*_2.5_ (m^3^)	2.79 (± 1.48) × 10^−5*^	21.8	0.35 (± 7.64 × 10^−3^)
*Vs2*_2.5_ (m^3^)	3.13 (± 1.54) × 10^−5*^	23.1	0.42 (± 0.01)
*Vs*_2.5_ (m^3^)	9.73 (± 3.72) × 10^−5*^	23.2	0.44 (± 0.01)

Trait abbreviations are provided in the section of Glossary. Significance level: ^***^ *p* ≤ 0.001; ^**^ *p* ≤ 0.01; ^*^ *p* ≤ 0.05; nonsignificant for all other *F*-values.

*H* ^2^ estimates for first-rotation growth traits ranged from 0.73 in *DBH*_15_ to 0.84 in *V*_2.5_, which tended to remain relatively stable over time and were generally higher than those of wood density (0.65 in *BD*_15_) and second-rotation growth traits (0.11–0.62; **Table 1**). The *H* ^2^ levels for the first rotation were in agreement with the previous report on earlier tree height (0.72–0.88 in *H* ^2^, except only *HT*_8_ at 0.40), diameter at breast height (0.76–0.84), and wood density (0.62 in *BD*_8_) of the same trial (Yang et al., 2018). According to Singh (2001), broad-sense heritability is considered low, medium, moderately high, and very high at values ≤ 0.40, 0.40–0.59, 0.60–0.79, and ≥ 0.80, respectively. Thus, the growth and wood density of the first rotation can be considered to be under moderately high or very high genetic control, while second-rotation growth traits tended to be under low to medium genetic control (except for *HTLBs*_1.5_, which had a moderately high *H* ^2^ of 0.62).

Despite the current study involving only a single cloned cross, the *H* ^2^ estimates obtained were comparable to those reported previously for multiple cloned families or crosses of *Eucalyptus*. For example, Reis et al. (2011) reported a clonal repeatability of over 0.74 for a 2-year-old volume of the first rotation in three trials of cloned *E*. *grandis* pure-species families and of *E*. *grandis* × *E*. *urophylla* interspecific crosses. Somewhat later, Amâncio et al. (2020) reported clonal repeatabilities of between 0.19 and 0.28 for 5.5-year-old coppice growth of the second rotation in five trials of cloned *E*. *grandis*, *E. saligna* Smith, and *E*. *urophylla* families and of *E*. *urophylla* × *E*. *grandis* crosses. Also, a similar magnitude of *H* ^2^ was found in a cloned single family of *P*. *taeda*, e.g., 0.79 and 0.75 for tree height and volume at the age of 6–7 years, respectively (Xiong et al., 2014).

Coppicing traits had *H* ^2^ between 0.35 in *GDs1*_0.5_ and 0.57 in *HTs2*_0.5_ (**Table 1**), revealing low to medium genetic control of these traits. In plantation management, coppice systems involving regeneration from stump sprouts after tree felling can be more economical (Crous and Burger, 2015; Hardiyanto et al., 2022) and sustainable (Zhou et al., 2017) than planting new stands. Coppicing traits are therefore important for guaranteeing plantation productivity and management profit. However, their genetic basis has been explored by only a few studies. Amâncio et al. (2020) found low broad-sense heritabilities (0.205–0.334) in post-cut survival of clonal trials in *E*. *grandis*, *E. saligna*, *E*. *urophylla*, and *E*. *urophylla* × *E*. *grandis*. Hernández et al. (2022) detected one and four quantitative trait loci related to numbers of post-fire basal resprouts and epicormic clusters, respectively, in *E. globulus* Labill. In combination with these studies, it can be highlighted that coppicing traits are characteristically under genetic control, at least in *Eucalyptus*, and the genetic effect may be low or medium depending on the trait.

Chlorophyll fluorescence and content traits fell in the range of 0.03–0.53 in *H* ^2^ (**Table 1**), indicating low to medium genetic control of the traits. Similar chlorophyll traits were investigated in earlier studies on forest trees under stress, e.g., high temperature in *Populus euphratica* Oliv. (Zhou et al., 2010) and elevated CO_2_ in *E*. *tereticornis* (Wujeska-Klausea et al., 2019), heritability including *H* ^2^ has rarely been reported. In other plants, broad-sense heritabilities for chlorophyll fluorescence traits have varied with trait and drought conditions from 0.40 to 0.74 in *Triticum turgidum* L. ssp. *durum* Desf. (Santos et al., 2021) and from 0.05 to 0.54 in *Sorghum bicolor* L (Ortiz and Salas-Fernandez, 2022). Also, *H* ^2^ fluctuations with light level have been reported for the quantum yield of photosystem II in *Arabidopsis thaliana* (L.) Heynh (Flood et al., 2016). Therefore, the magnitude of *H* ^2^ estimates for chlorophyll-related traits may depend on species, population, and/or environmental conditions. In this respect, the present work provides new information about the genetic control of photosynthetic traits for woody plants.

### 3.3 Phenotypic and additive genetic correlations

**Figure 1:**
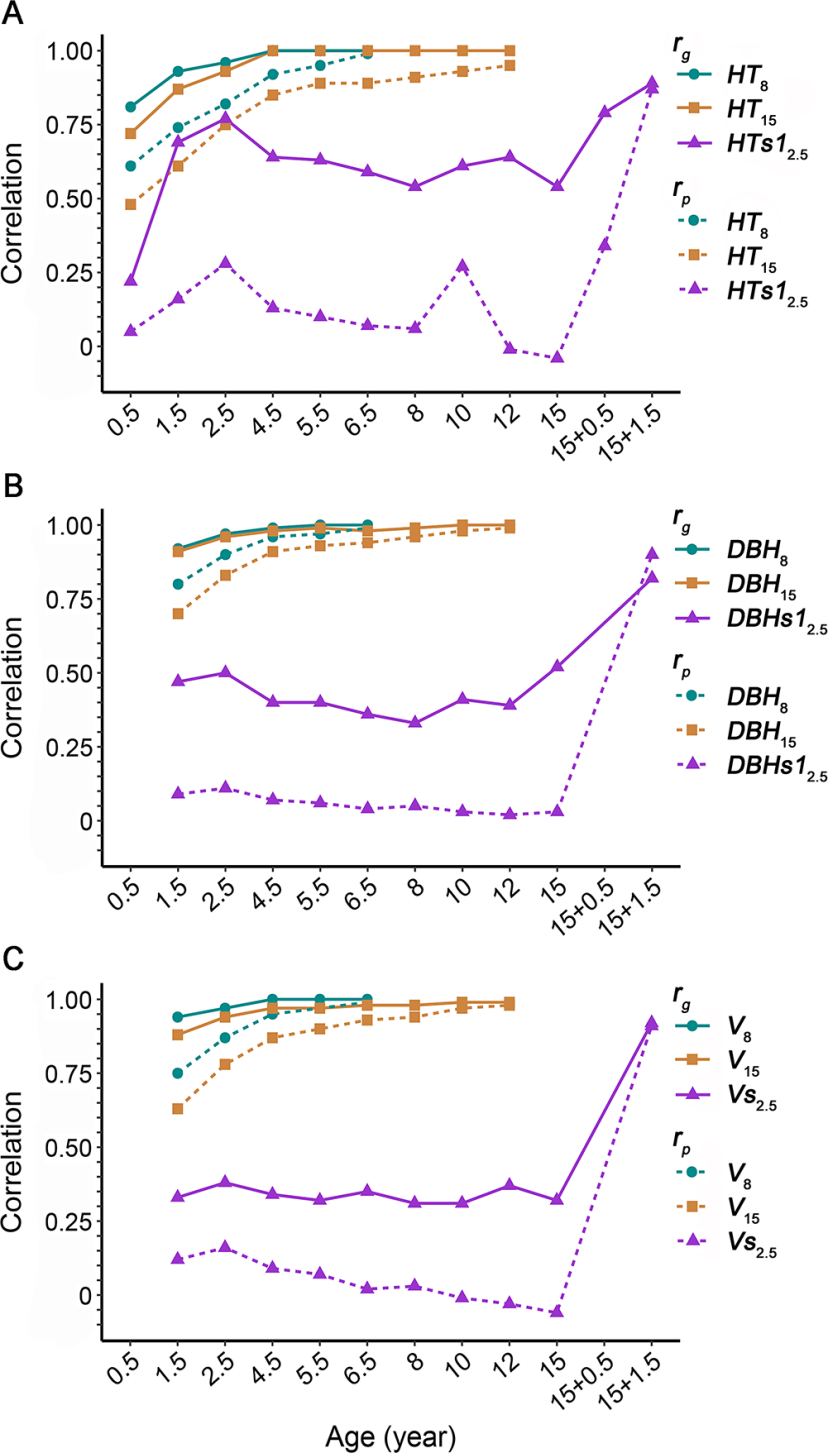
Phenotypic correlations ( $r_{p}$ ) and additive genetic correlations ( $r_{g}$ ) for growth at ages 8 and 15 years in the first rotation and age 2.5 years in the second rotation with earlier respective growth traits. **(A)** Tree height (*HT*). **(B)** Diameter at breast height (*DBH*). **(C)** Volume (*V*). In the legend, numerals following the trait abbreviation indicate the tree or sprout age. For growth at age 2.5 years in the second rotation, *HT* and *DBH* of the higher sprout and total *V* of two sprouts (only one in a few cases) per stump were used for calculating correlations. Trait abbreviations are provided in the section of Glossary.

Trait–trait correlations $r_{p}$ and $r_{g}$ are presented in **Supplementary Table 3**. The first-rotation growth traits showed consistently favorable and positive $r_{p}$ and $r_{g}$ , ranging from 0.31 to 0.99 and from 0.66 to 1.00, respectively, whereas wood properties had $r_{p}$ and $r_{g}$ between −0.46 and 0.43 and between −0.80 and 0.82, respectively. In terms of age-age correlations, the earlier growth traits appeared to correlate increasingly with time with that of the final age. For instance, $r_{p}$ and $r_{g}$ of *HT*_15_, *DBH*_15_, and *V*_15_ increased from 0.48 and 0.72 (with *HT*_0.5_) to 0.95 and 1.00 (with *HT*_12_), 0.70 and 0.91 (with *DBH*_1.5_) to 0.99 and 1.00 (with *DBH*_12_), and 0.63 and 0.88 (with *V*_1.5_) to 0.98 and 0.99 (with *V*_12_), respectively, and remained relatively high with the respective counterpart traits after age 2.5 (more than 0.75 and 0.93 in $r_{p}$ and $r_{g}$ , respectively; **Figure 1**). Meanwhile, moderate age-age correlations were observed in wood basic density, with $r_{p}$ and $r_{g}$ of 0.23 and 0.68 between *BD*_15_ and *BD*_8_, respectively. Moreover, for correlations between growth and wood properties, growth traits showed generally positive $r_{p}$ and $r_{g}$ with *BD*_8_, *CC*_8_, and *BD*_15_, negative $r_{p}$ and $r_{g}$ with *HC*_8_ and *S*/*G*_8_, but negative $r_{p}$ and positive $r_{g}$ with *LC*_8_. Similar correlation patterns were reported earlier for the same cross over three environments at age 8 (Yang et al., 2018).

The high correlations between growth traits obtained in the current study generally concur with some earlier studies on *Eucalyptus* clones, e.g., genotypic correlations being 0.85–0.90 and 0.94–0.96 between height and diameter in clones of *E. camaldulensis* (ages 3 and 5; Kien et al., 2010) and *E. globulus* (ages 4 and 12; Costa e Silva et al., 2013). Though correlations between wood traits for *Eucalyptus* clones were available in a couple of reports (Kien et al., 2010; Gallo et al., 2018; Bouvet et al., 2020), only Gallo et al. (2018) analyzed merely a pair of wood traits comparable to this study, who also found a negative genotypic correlation (−0.54) between wood basic density and Klason lignin content in *E. dunnii* Maiden clones. Moreover, similar positive and negative genetic correlations were frequently observed between wood property traits in seedling-based family tests in *Eucalyptus*, e.g., 0.36 between seedling wood basic density and cellulose content in *E. nitens* Deane & Maiden (age 9; Hamilton et al., 2009) and − 0.31 between *S*/*G* and lignin content in *E. globulus* (age 16; Stackpole et al., 2010). This may indicate a small effect of propagule type (seedling versus vegetatively propagated clone) on trait-trait genetic correlations (Costa e Silva et al., 2013). Furthermore, weak genetic correlations of clonal growth with lignin content detected here (0.03–0.06 at age 8) are in accordance with those observed in clones of *E. dunnii* (− 0.19 to − 0.13 at age 3.5; Gallo et al., 2018) and *E*. *urophylla* × *E*. *grandis* (0.238 at 55 months of age; Bouvet et al., 2020). However, the relatively high positive correlations between growth and basic density (0.67–0.82 and 0.44–0.67 at ages 8 and 15, respectively) are opposite to those reported for *E*. *grandis* (− 0.06 to 0.06 and − 0.08 to 0.16 in $r_{g}$ of basic density with height and mean annual increment, respectively, across three sites at age 6; Osorio et al., 2003), *E. camaldulensis* (0.17 and − 0.16 between height and basic density and 0.21 and 0.07 between *DBH* and basic density for two sites at age 5; Kien et al., 2010), and *E. dunnii* (− 0.04, − 0.01 and 0.02 in $r_{g}$ of basic density with *DBH*, height, and mean annual increment, respectively, at age 3.5; Gallo et al., 2018). This may suggest the variation of such correlations depends on both population and environment.

For coppicing traits, trait-trait correlations were positive and generally high, with $r_{p}$ of 0.17–0.91 and $r_{g}$ of 0.65–1.00. Moreover, coppicing traits *NS*_0.5_ and *CP*_0.5_ had moderate positive $r_{p}$ and $r_{g}$ with the first-rotation final growth, namely, 0.28–0.37 for $r_{p}$ and 0.47–0.67 for $r_{g}$ with *HT*_15_, *DBH*_15_, and *V*_15_. Meanwhile, *HTs1*_0.5_, *HTs2*_0.5_, *GDs1*_0.5_, and *GDs2*_0.5_ had very weak $r_{p}$ (− 0.01 to 0.05) but moderately low positive $r_{g}$ (0.18–0.43) with those growth traits. Coppicing traits were weakly correlated with the wood trait *BD*_15_, with $r_{p}$ and $r_{g}$ being − 0.03 to 0.13 and − 0.33 to 0.15, respectively. This study represents by far the only attempt in *Eucalyptus* to survey the correlations between coppicing ability and earlier-rotation traits, which could be helpful for understanding the relationship between these traits.

Chlorophyll fluorescence and content traits were correlated variably with each other, with $r_{p}$ and $r_{g}$ being − 0.83 to 0.98 and − 0.96 to 0.99, respectively. Their $r_{p}$ estimates with the traits observed earlier were consistently very weak while those of $r_{g}$ varied notably with trait, e.g., − 0.17 to 0.28, − 0.12 to 0.10, and − 0.14 to 0.12 for $r_{p}$ and − 0.98 to 1.00, − 0.41 to 0.99, and − 0.90 to 1.00 for $r_{g}$ with first-rotation growth, wood property, and coppicing traits, respectively. The majority of $r_{g}$ estimates were accompanied by large standard errors, suggesting non-significance of these correlations. Specifically, the negative $r_{p}$ between *F*_0_ and *Y(II)* (−0.48) and between *Y(II)* *′* and *NPQ* (− 0.30) is in line with the observation that decline of *F*_v_/*F*_m_ [*Y(II)*] and *F*_v_′/*F*_m_′ [*Y(II)* *′*] was associated with increased *F*_0_ and *NPQ* in two eucalypt clones under Cd stress (Pietrini et al., 2015). On the other hand, the very weak $r_{p}$ observed between *Y(II)* and *Y(II)* *′* (− 0.03) was in sharp contrast to that of a highly significant correlation (0.42, *p* < 0.001) in *S. bicolor* under normal conditions (Ortiz and Salas-Fernandez, 2022). Nevertheless, correlations between chlorophyll fluorescence, chlorophyll content, and other trait types have rarely been reported from forest trees, and our results will provide valuable information in this context.

Except for *BAs*_1.5_ and *HTLBs*_1.5_, 1.5- and 2.5-year-old growth traits of the second rotation showed generally high positive $r_{p}$ and $r_{g}$ with each other, demonstrating the strong correlations between growth traits such as sprout height, diameter at breast height, volume, and crown projected area. For instance, irrespective of *BAs*_1.5_ and *HTLBs*_1.5_, *Vs*_1.5_ had $r_{p}$ ranging from 0.25 (with *NBs*_1.5_) to 0.91 (with *Vs*_2.5_) and $r_{g}$ ranging from 0.47 (with *NBs*_1.5_) to 1.00 (with *Vs1*_1.5_ and *CPAs*_1.5_). In contrast, *BAs*_1.5_ had consistently weak $r_{p}$ (0.02–0.14) and $r_{g}$ (− 0.66 to 0.70 with high stand errors) with the other traits, while *HTLBs*_1.5_ had low to intermediate $r_{p}$ (− 0.11 to 0.54) and $r_{g}$ (− 0.51 to 0.38 with relatively high stand errors). Similar weak $r_{g}$ of branch angle with tree height (− 0.24) and volume (− 0.02) were noted in *P. taeda* clones (Xiong et al., 2014). In particular, the final volume *Vs*_2.5_ had very weak $r_{p}$ but moderate positive $r_{g}$ with earlier volume of the first rotation such as *V*_8_ and *V*_15_ (0.03 and − 0.06 for $r_{p}$ and 0.31 and 0.32 for $r_{g}$ , respectively). The very weak $r_{p}$ in volume between the first and second rotations was in discrepancy with those observed for 5.5-year-old volume across rotations in *Eucalyptus* clones (more than 0.71; Amâncio et al., 2020). Such a discrepancy may reflect that across-rotation correlations are population and/or age-specific.

### 3.4 Multitrait selection, relative genetic gain, and selection efficiency

Multitrait SI calculations were accomplished with the following four scenarios: 10.90*V*_8_ + 11.96*BD*_8_ + 0.50*CC*_8_ (scenario 1), 2.12*V*_15_ + 10.33*BD*_15_ (scenario 2), 11.27*V*_8_ + 18.48*BD*_8_ + 0.22*CC*_8_ + 0.50*CP*_0.5_ + 22.31*Vs*_2.5_ (scenario 3), and 3.50*V*_15_ + 4.61*BD*_15_ + 0.18*CP*_0.5_ + 12.01*Vs*_2.5_ (scenario 4). This indicates that the relative economic weight of an objective trait can vary with the traits involved. A total of 48, 44, 41, and 41 clones were selected under scenarios 1–4, resulting in selection differentials of 4.14, 3.62, 7.00, and 3.40, respectively (**Supplementary Table 4**). For MGIDI selection, scenario 1′, 2′, 3′, and 4′ involved the same traits as the counterpart scenarios 1, 2, 3, and 4, respectively. Only factors with eigenvalues of more than 1.00 were included, leading to one, one, two, and two factors with an accumulated variance of 56.5%, 60.0%, 60.1%, and 66.8% in scenarios 1′–4′, respectively (**Table 2**). Selection differentials ranged from − 1.55 to − 1.33, with the smaller value being the better. Scenarios 1′–4′ shared 35 (72.9%), 39 (88.6%), 24 (58.5%), and 35 (85.4%) selected clones, respectively, with the counterpart SI scenario (**Supplementary Table 4**). In addition, single-trait selection for *V*_1.5_, *V*_2.5_, *V*_4.5_, *V*_5.5_, *V*_6.5_, *V*_8_, *BD*_8_, *CC*_8_, *V*_10_, *V*_12_, *V*_15_, *DB*_15_, *CP*_0.5_, *Vs*_1.5_, and *Vs*_2.5_ resulted in 0.003–0.52 of selection differential in *BV*, with 60–41 clones selected (**Supplementary Tables 5**, **6**).

**Table 2:** Factors retained in the multitrait genotype–idiotype distance index (MGIDI) analysis along with their eigenvalues, explained variances, and accumulative variances.

Trait	Scenario 1′: *V*_8_+*BD*_8_+*CC*_8_	Scenario 2′: *V*_15_+*BD*_15_	Scenario 3′: *V*_8_+*BD*_8_+*CC*_8_+*CP*_0.5_+*Vs*_2.5_		Scenario 4′: *V*_15_+*BD*_15_+*CP*_0.5_+*Vs*_2.5_	
	Factor 1	Factor 1	Factor 1	Factor 2	Factor 1	Factor 2
*V*_8_	− 0.76	–	− 0.79	− 0.14	–	–
*BD*_8_	− 0.87	–	− 0.85	0.07	–	–
*CC*_8_	− 0.60	–	− 0.47	− 0.16	–	–
*V*_15_	–	0.77	–	–	0.79	0.24
*BD*_15_	–	0.77	–	–	0.30	0.78
*CP*_0.5_	–	–	− 0.46	− 0.60	0.79	− 0.18
*Vs*_2.5_	–	–	0.04	− 0.90	0.41	− 0.69
Eigenvalue	1.69	1.20	1.97	1.03	1.50	1.17
Variance explained (%)	56.5	60.0	39.4	20.7	37.5	29.3
Accumulative variance (%)	–	–	–	60.1	–	66.8

Trait abbreviations are provided in the section of Glossary.

**Figure 2:**
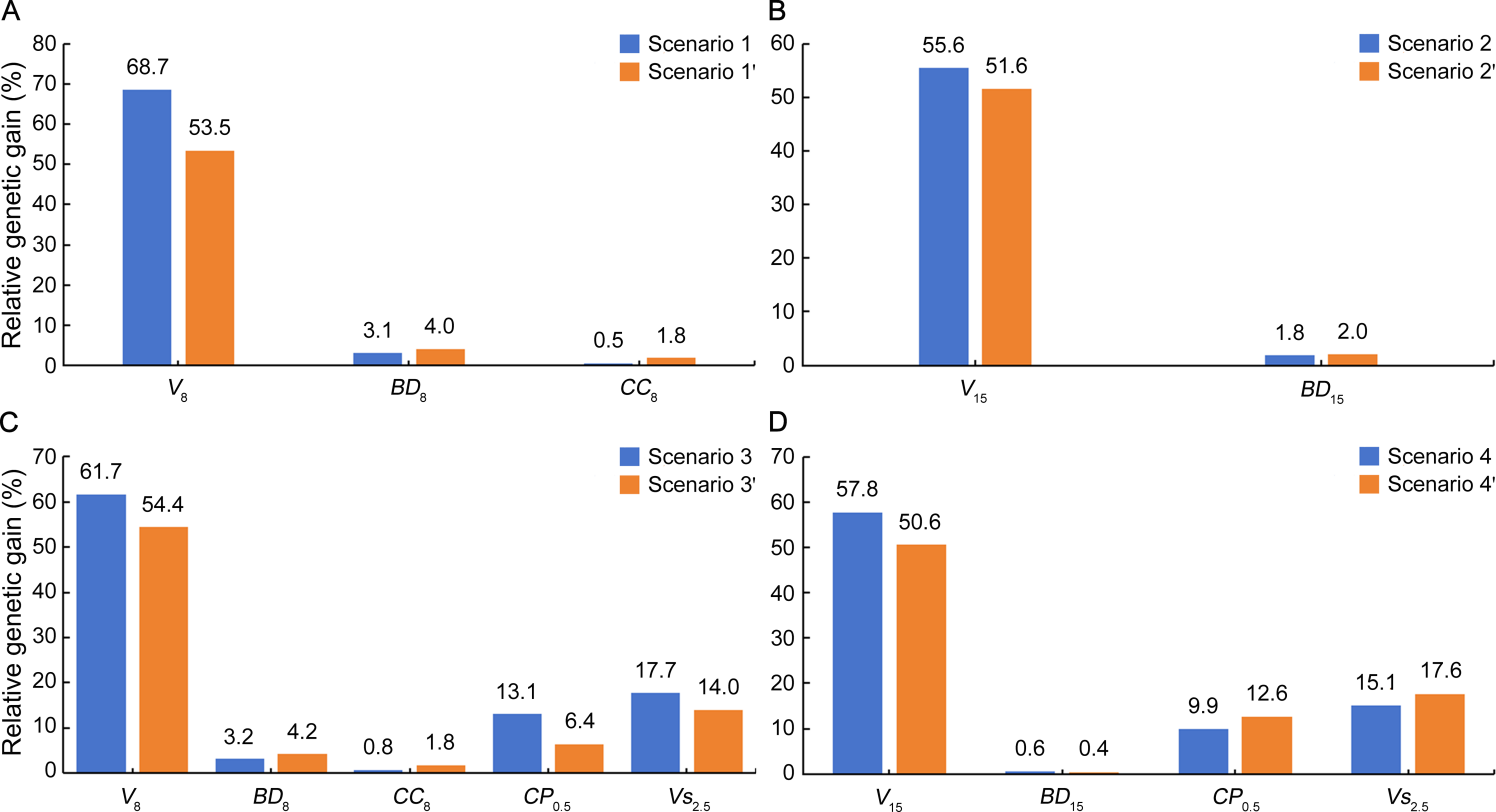
Relative genetic gain (*RG*, %) for multitrait selection scenarios targeting traits in a cloned *E. urophylla* × *E. tereticornis* cross. Trait abbreviations are provided in the section of Glossary.

*RG* estimates for all the traits involved in multitrait selection scenarios were positive (**Figure 2**), indicating the usefulness of all the scenarios for the combined selection of these traits. Of the trait types, growth of the first rotation (*V*_8_ and *V*_15_) showed consistently the highest *RG* (50.6%–68.7%) as compared to wood properties (*BD*_8_, *CC*_8_, and *BD*_15_; 0.46%–4.2%), coppicing (*CP*_0.5_; 6.4%–13.1%), and growth of the second rotation (*Vs*_2.5_; 14.0–17.7%). These estimates are extremely high for growth but comparable for wood properties relative to genetic gains reported by others for the selection of *Eucalyptus* clones, e.g., 7.5%–20.4% for volume and − 0.3% to 0.4% for cellulose content in *E. urophylla* × *E. grandis* clones selected using an equal emphasis SI method (Bouvet et al., 2020). In terms of *RG* comparison between selection methods, SI was always preferred for a higher *RG* in the first-rotation growth *V*_8_ and *V*_15_ whereas MGIDI was more favorable for wood properties *BD*_8_, *CC*_8_, and *BD*_15_ except for *BD*_15_ with slightly lower *RG* in scenario 4′. In addition, single-trait selections resulted in *RG* of 45.1%–79.1% in the first-rotation growth, 3.4%–4.6% in wood properties, 16.1% in coppicing potential, and 38.8% in *Vs*_2.5_ (**Supplementary Table 5**).

**Figure 3:**
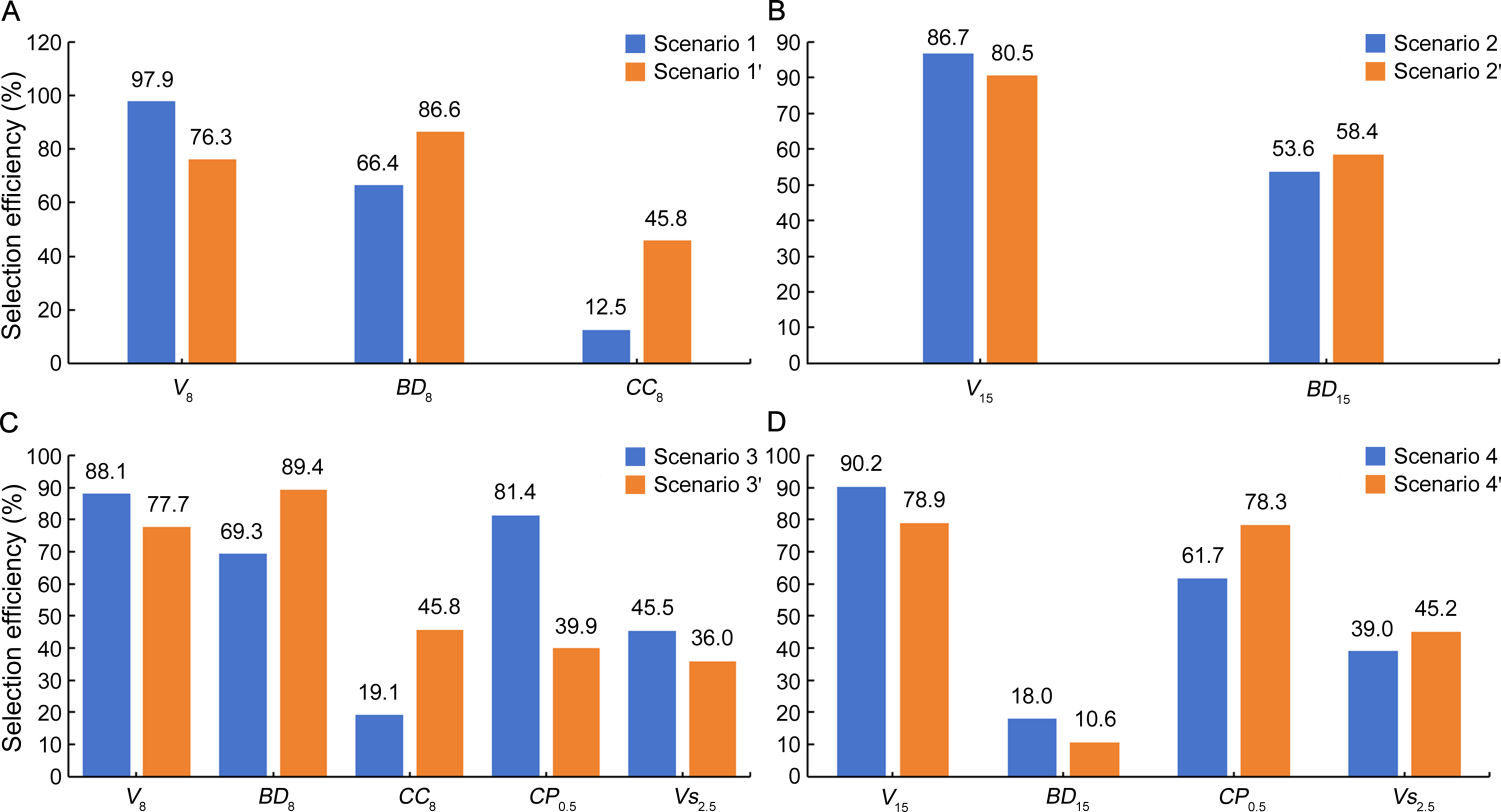
Selection efficiency (*E*, %) of multitrait selection scenarios relative to each single-trait selection in a cloned *E. urophylla* × *E. tereticornis* cross. Trait abbreviations are provided in the section of Glossary.

In accordance with *RG* estimates, *E* values for all the multitrait selection scenarios relative to each single-trait selection were also positive (**Figure 3**) but varied largely from 10.6% (*BD*_15_ with MGIDI) to 97.9% (*V*_8_ with SI). As compared to the SI method, MGIDI showed somewhat lower *E* in first-rotation growth *V*_8_ and *V*_15_ but higher *E* in wood properties *BD*_8_, *CC*_8_, and *BD*_15_ except for *BD*_15_ with slightly lower *E* in scenario 4′. Similarly, in *Avena strigosa* S., desirable selection efficiency varied with the selection method (SI or MGIDI) for the same trait (Klein et al., 2023). In addition, *E* of earlier single-trait selections in volume was relatively high for *V*_8_ (70.7%–95.8%), low to moderately high for *V*_15_ (33.0%–76.9%), *BD*_8_ (31.4%–51.0%), and *CP*_0.5_ (27.3%–46.5%), very low to low for *CC*_8_ (− 0.3% to 0.3%), *Vs*_1.5_ (− 16.1% to 7.4%), and *Vs*_2.5_ (− 5.1% to 12.8% with the only exception of 88.5% by *Vs*_1.5_), and extremely low for *BD*_15_ (− 250.9% to − 69.7%; **Supplementary Table 5**). Specifically, single-trait selections in *V*_8_, *V*_8_, and *V*_15_ gave rise to *E* of 42.1%, 0.04%, and − 120.6% for wood properties *BD*_8_, *CC*_8_, and *BD*_15_, respectively (**Supplementary Table 5**), being somewhat less than those of multitrait selections. Meanwhile, single-trait selections in volume at ages 2.5 and 6.5 could have brought about *E* more than 80% and 70% for *V*_8_ and *V*_15_, respectively, indicating the possibility of effective volume selection at less than half-rotation age.

### 3.5 Implications for eucalypt clonal breeding

Clonal tests usually focus on the first rotation performance in forest trees such as eucalypts (Lambeth et al., 1994; Osorio et al., 2001, 2003; Reis et al., 2011; Amâncio et al., 2020; Romão et al., 2023) and poplars (Marron and Ceulemans, 2006; Rönnberg-Wästljung et al., 2022). This can be meaningful for estimating genetic parameters and evaluating clonal performance within a single rotation, especially for those plantation trees regenerated merely through means of replanting. However, in regard to across-rotation trait variability and management regime shifts, information only on the first rotation may not be enough for trees in plantation systems amenable to coppice regeneration. Here, the weak or moderate $r_{p}$ and $r_{g}$ in volume between rotations (e.g., $r_{p}$ and $r_{g}$ between *V*_15_ and *Vs*_2.5_ being − 0.06 and 0.32, respectively; **Figure 1C**, **Supplementary Table 3**), coupled with remarkably poor selection efficiency in volume across rotations (e.g., 0.7% of *V*_15_ on *Vs*_2.5_; **Supplementary Table 5**) and relatively low proportion of common clones between single- and across-rotation multitrait selection scenarios (e.g., 56.1% between scenarios 1′ and 4′; **Supplementary Table S4**), may justify the across-rotation growth variability and the necessity of across-rotation investigation. Also, across-rotation growth variability was noted in clones of other eucalypt taxa, which may be related to management regime shifts such as retention of more than one sprout per tree (Amâncio et al., 2020).

Both SI and MGIDI methods are effective in guaranteeing desirable positive *RG* and *E* for all the selection traits (**Figures 2**, **3**), demonstrating the potential for simultaneous improvement of the traits involved. Nevertheless, the two methods differed more or less in selected clones (**Supplementary Table 4**). Such difference in selected genotypes was also noticed between SI and MGIDI in *Zea mays* L. (Yue et al., 2022) and *Avena strigosa* S (Klein et al., 2023). As MGIDI uses a factor analysis process free from such economic coefficient weightings and multicollinearity issues that SI relies on, it brings more balanced genetic gains (Klein et al., 2023). This seems to be the case for *E. urophylla* × *E*. *tereticornis* clones, as each of the MGIDI selection scenarios shows a narrower range of *RG* (also *E*) values than the respective SI scenarios (**Figures 2**, **3**). Therefore, neither of the two methods can fit all multitrait selection circumstances in our eucalypt hybrid clones, and the choice of method depends on the tradeoff of expected genetic gains among the traits involved. If a higher *RG* (also *E*) is expected for a specific target trait, the SI method with a higher weighting of the trait would be preferred. Moreover, as a factor analysis tends to group positively and negatively correlated variables into the same factor (Joliffe and Morgan, 1992), MGIDI is limited in selection for two traits in opposite selection gains (Olivoto et al., 2022).

Early selection is attractive in tree breeding because selection prior to rotation age can capture genetic gains sooner and therefore increase gains achievable per unit of time (White et al., 2007). The efficiency of early selection is affected by the heritability of a measured trait and its genetic correlation with a target trait (Jansson et al., 2003; White et al., 2007). In the present study, like many previous reports for eucalypts (e.g. Osorio et al., 2001, 2003; He et al., 2012; Araujo et al., 2021; Miranda et al., 2024), the heritability estimates of volume are similar magnitude over ages within the first rotation, and age–age genetic correlations should have a more pronounced influence on selection efficiency. In consideration of those strong correlations between earlier and rotation ages (age 8 for veneer and pulping and 15 for large-size timber purposes), together with the favorable *E* estimates (**Supplementary Table 5**), half-rotation or somewhat earlier age can be determined for early growth selection, that is, age 4.5 or 2.5 for veneer and pulp usages and age 8.5 or 6.5 for large-diameter timber production. Similar early selection timelines have previously been proposed for *Eucalyptus* clones (Osorio et al., 2003; Massaro et al., 2010; He et al., 2012; Moraes et al., 2014; Yang et al., 2018).

The finding of significant differences between ramet means and ortet values for most traits, especially those involved in multitrait selection such as *V*_8_, *V*_15_, and *CP*_0.5_ (*p* ≤ 0.001 or 0.01; **Supplementary Table 2**), can also have implications for clonal breeding. Firstly, seedling-based genetic information such as heritability (or repeatability), genetic correlation, and breeding value cannot be used for clonal selection. This is true particularly for low-heritable traits, as seedling-clone correlation depends largely on the magnitude of genetic control of the trait concerned (Costa e Silva et al., 2013). Secondly, direct clonal selection may be the desirable option rather than the inclusion of prior seedling progeny test data. Also, van den Berg et al. (2015) questioned the feasibility of using prior seedling selection for *E. grandis* and *E. urophylla* clonal testing. Thirdly, the gap between seedling and clonal selections may be bridged by molecular marker-assisted tools such as genomic selection (GS). GS offers an efficient tool to screen a large number of genotypes, reduce the time for selection of an operational clone, and consequently increase genetic gains (Balocchi et al., 2023). GS has been recently conducted for *Eucalyptus* clone selection in a number of eucalypt breeding programs (Torres-Dini et al., 2016; Durán et al., 2017; Balocchi et al., 2023). If a GS model is validated efficiently for predicting performance in a clonal population, it can be applied to screen much larger seedling (also clonal) populations of the kind at a considerably low selection intensity. Consequently, only a small number of clones (say, 30 to 50) identified by GS need to be field validated, and the genetic gain will increase in the clonal deployment population (Balocchi et al., 2023).

## 4 Conclusion

This study provides novel information on across-rotation genetic parameters and multitrait selection methods based on a cloned *E. urophylla* × *E. tereticornis* cross. The high progeny phenotypic variability suggests the potential of clone selection within a single cross. The relatively high *H* ^2^ estimates for some traits reveal high genetic control of such traits. The generally weak or moderate correlations in growth between rotations may justify the across-rotation growth variability and the necessity of across-rotation investigation. Neither the SI nor the MGIDI method can fit all multitrait selection circumstances, and the choice of method depends on the tradeoff of expected genetic gains among the traits involved. Half-rotation or somewhat earlier age can be used for the early selection of growth traits. Direct clonal selection may be desirable rather than the inclusion of prior seedling progeny tests, and seedling-based selection may be integrated using molecular marker-assisted approaches. The results can have important implications for eucalypt clone breeding and management, including the necessity of across-rotation investigation, evaluation of multitrait selection options, determination of selection ages, and the possible use of seedling deployment population.

## Data Availability

The original contributions presented in the study are included in the article/**Supplementary Material**. Further inquiries can be directed to the corresponding authors.

## Glossary

MGIDI: multitrait genotype–idiotype distance index
*H* ^2^: clonal repeatability
*RG*: relative genetic gain (%)
*E*: selection efficiency (%)
*HT*_n_: *HT*_0.5_, *HT*_1.5_, *HT*_2.5_, *HT*_4.5_, *HT*_5.5_, *HT*_6.5_, *HT*_8_, *HT*_10_, *HT*_12_, and *HT*_15_, tree height (m) at age of around 0.5, 1.5, 2.5, 4.5, 5.5, 6.5, 8, 10, 12, and 15 years of the first rotation, respectively
*DBH*_n_: *DBH*_1.5_, *DBH*_2.5_, *DBH*_4.5_, *DBH*_5.5_, *DBH*_6.5_, *DBH*_8_, *DBH*_10_, *DBH*_12_, and *DBH*_15_, diameter at breast height (cm) at age of around 1.5, 2.5, 4.5, 5.5, 6.5, 8, 10, 12, and 15 years of the first rotation, respectively
*BD*_15_: 15-year-old wood basic density (g/cm^3^) of the first rotation
*BD*_8_: *CC*_8_, *HC*_8_, *LC*_8_, and *S/G*_8_, 8-year-old wood basic density (g/cm^3^), cellulose content (%), hemicellulose content (%), lignin content (%), and syringyl-to-guaiacyl lignin ratio of the first rotation, respectively
*V*_n_: *V*_1.5_, *V*_2.5_, *V*_4.5_, *V*_5.5_, *V*_6.5_, *V*_8_, *V*_10_, *V*_12_, and *V*_15_, volume (m^3^) at age of around 1.5, 2.5, 4.5, 5.5, 6.5, 8, 10, 12, and 15 years of the first rotation, respectively
NS_0.5_: *HTs1*_0.5_, *HTs2*_0.5_, *GDs1*_0.5_, *GDs2*_0.5_, and *CP*_0.5_, number of sprouts, height of the highest sprout (m), height of the second highest sprout (m), ground diameter of the highest sprout (cm), ground diameter of the second highest sprout (cm), and coppicing potential measured at 0.5 year after felling, respectively
*F*_0_ *F*_m_, and *F*_v_ (= *F*_m_–*F*_0_): initial, maximal, and variable leaf chlorophyll fluorescence after dark adapting, respectively
*Y(II)*(=*F*_v_/*F*_m_): maximal quantum yield of photosystem II after dark adaptation
*F*_s_ *F*_m_′, and *F*_v_′ (=*F*_s_): steady-light, maximal, and variable leaf chlorophyll fluorescence after light adaptation, respectively
Y(*II*)′ (= *F*_v_′/*F*_m_′): maximal quantum yield of photosystem II after light adaptation
*ETR*: photosynthetic electron transport rate under light-adaptation
*NPQ*: nonphotochemical quenching
*SPADR*: SPAD (Spectrum Technologies Inc.) reading
*HTs1*_1.5_: *HTs1*_2.5_, *DBHs1*_1.5_, *DBHs1*_2.5_, *Vs1*_1.5_, and *Vs1*_2.5_, 1.5- and 2.5-year-old height (m), diameter at breast height (cm), and volume (m^3^) of the highest sprout for the second rotation, respectively
*HTs2*_1.5_: *HTs2*_2.5_, *DBHs2*_1.5_, *DBHs2*_2.5_, *Vs2*_1.5_, and *Vs2*_2.5_, 1.5- and 2.5-year-old height (m), diameter at breast height (cm), and volume (m^3^) of the second highest sprout for the second rotation, respectively
*Vs*_1.5_: the sum of *Vs1*_1.5_ and *Vs2*_1.5_
*Vs*_2.5_: the sum of *Vs1*_2.5_ and *Vs2*_2.5_
*BAs*_1.5_: *HTLBs*_1.5_, *NBs*_1.5_, *CWs1*_1.5_, *CWs2*_1.5_, *CLs*_1.5_, and *CPAs*_1.5_, branch angle (^o^), height of the lowest live branch (m), number of branches, crown width of the highest sprout (m), crown width of the second highest sprout (m), crown length (m), and crown projected area (m^2^) of the 1.5-year-old sprouts per stump for the second rotation, respectively
SD: standard deviation
CV: coefficient of variation (%)
ANOVA: analysis of variance
CV_g_: coefficient of genetic variation (%)
*r_p_*: phenotypic correlation
*r_g_*: additive genetic correlation
SE: standard error
BLUP: best linear unbiased prediction
*BV*: breeding value
SI: selection index
GS: genomic selection.
