# Supplementary material for: Across-rotation genetic analysis and multitrait selection in a cloned cross of Eucalyptus urophylla × E. tereticornis
Source: Front Plant Sci. 2025 Jun 3;16:1553819. doi: 10.3389/fpls.2025.1553819 (PMC12172632; doi:10.3389/fpls.2025.1553819)
Supplement: Supplementary file 1 [file Table1.docx]

Supplementary Material: Supplementary Tables 1, 2, and 5

*Abbreviations:* MGIDI, multi-trait genotype-idiotype distance index; *H*^2^, clonal repeatability; *RG*, relative genetic gain (%); *E*, selection efficiency (%); *HT*_0.5_, *HT*_1.5_, *HT*_2.5_, *HT*_4.5_, *HT*_5.5_, *HT*_6.5_, *HT*_8_, *HT*_10_, *HT*_12_, and *HT*_15_, tree height (m) at age of around 0.5, 1.5, 2.5, 4.5, 5.5, 6.5, 8, 10, 12, and 15 years of the first rotation, respectively; *DBH*_1.5_, *DBH*_2.5_, *DBH*_4.5_, *DBH*_5.5_, *DBH*_6.5_, *DBH*_8_, *DBH*_10_, *DBH*_12_, and *DBH*_15_, diameter at breast height (cm) at age of around 1.5, 2.5, 4.5, 5.5, 6.5, 8, 10, 12, and 15 years of the first rotation, respectively; *BD*_15_, 15-year-old wood basic density (g/cm^3^) of the first rotation; *BD*_8_, *CC*_8_, *HC*_8_, *LC*_8_, and *S/G*_8_, 8-year-old wood basic density (g/cm^3^), cellulose content (%), hemicellulose content (%), lignin content (%), and syringyl-to-guaiacyl lignin ratio of the first rotation, respectively; *V*_1.5_, *V*_2.5_, *V*_4.5_, *V*_5.5_, *V*_6.5_, *V*_8_, *V*_10_, *V*_12_, and *V*_15_, volume (m^3^) at age of around 1.5, 2.5, 4.5, 5.5, 6.5, 8, 10, 12, and 15 years of the first rotation, respectively; *NS*_0.5_, *HTs1*_0.5_, *HTs2*_0.5_, *GDs1*_0.5_, *GDs2*_0.5_, and *CP*_0.5_, number of sprouts, height of the highest sprout (m), height of the second highest sprout (m), ground diameter of the highest sprout (cm), ground diameter of the second highest sprout (cm), and coppicing potential measured at 0.5 year after felling, respectively; *F*_0_, *F*_m_, and *F*_v_ (= *F*_m_ – *F*_0_), initial, maximal, and variable leaf chlorophyll fluorescence after dark adapting, respectively; *Y(II)* (= *F*_v_/*F*_m_), maximal quantum yield of photosystem II after dark adaptation; *F*_s_, *F*_m_', and *F*_v_' (= *F*_m_' – *F*_s_), steady-light, maximal, and variable leaf chlorophyll fluorescence after light adaptation, respectively; *Y(II)*' (= *F*_v_'/*F*_m_'), maximal quantum yield of photosystem II after light adaptation; *ETR*, photosynthetic electron transport rate under light-adaptation; *NPQ*, non-photochemical quenching; *SPADR*, SPAD (Spectrum Technologies Inc.) reading; *HTs1*_1.5_, *HTs1*_2.5_, *DBHs1*_1.5_, *DBHs1*_2.5_, *Vs1*_1.5_, and *Vs1*_2.5_, 1.5- and 2.5-year-old height (m), diameter at breast height (cm), and volume (m^3^) of the highest sprout for the second rotation, respectively; *HTs2*_1.5_, *HTs2*_2.5_, *DBHs2*_1.5_, *DBHs2*_2.5_, *Vs2*_1.5_, and *Vs2*_2.5_, 1.5- and 2.5-year-old height (m), diameter at breast height (cm), and volume (m^3^) of the second highest sprout for the second rotation, respectively; *Vs*_1.5_, the sum of *Vs1*_1.5_ and *Vs2*_1.5_; *Vs*_2.5_, the sum of *Vs1*_2.5_ and *Vs2*_2.5_; *BAs*_1.5_, *HTLBs*_1.5_, *NBs*_1.5_, *CWs1*_1.5_, *CWs2*_1.5_, *CLs*_1.5_, and *CPAs*_1.5_, branch angle (^o^), height of the lowest live branch (m), number of branches, crown width of the highest sprout (m), crown width of the second highest sprout (m), crown length (m), and crown projected area (m^2^) of the 1.5-year-old sprouts per stump for the second rotation, respectively; SD, standard deviation; CV, coefficient of variation (%); ANOVA, analysis of variance; CV_g_, coefficient of genetic variation (%); $r_{p}$, phenotypic correlation; $r_{g}$, additive genetic correlation; SE, standard error; BLUP, best linear unbiased prediction; *BV*, breeding value; SI, selection index; GS, genomic selection.

**Supplementary Table 1.** Descriptive statistics for 54 traits investigated in a cloned *Eucalyptus* *urophylla* × *E*. *tereticornis* cross.

| **Trait** | **N** | **Mean (± SD)** | **Minimum** | **Maximum** | **Coefficient of variation (%)** |
| --- | --- | --- | --- | --- | --- |
| *V*_1.5_ (m^3^) | 400 | 0.02 (± 0.01) | 1.73 × 10^−4^ | 0.04 | 39.1 |
| *V*_2.5_ (m^3^) | 391 | 0.03 (± 0.01) | 5.76 × 10^−5^ | 0.07 | 41.9 |
| *V*_4.5_ (m^3^) | 374 | 0.08 (± 0.04) | 1.93 × 10^−3^ | 0.22 | 53.8 |
| *V*_5.5_ (m^3^) | 367 | 0.11 (± 0.06) | 3.10 × 10^−3^ | 0.32 | 57.1 |
| *V*_6.5_ (m^3^) | 324 | 0.14 (± 0.08) | 0.01 | 0.40 | 52.7 |
| *V*_8_ (m^3^) | 318 | 0.17 (± 0.09) | 0.01 | 0.51 | 52.8 |
| *HT*_10_ (m) | 298 | 22.41 (± 2.98) | 13.00 | 27.35 | 13.3 |
| *DBH*_10_ (cm) | 317 | 16.91 (± 4.17) | 6.70 | 28.73 | 24.7 |
| *V*_10_ (m^3^) | 298 | 0.26 (± 0.13) | 0.03 | 0.77 | 51.5 |
| *HT*_12_ (m) | 286 | 22.73 (± 3.06) | 13.00 | 27.50 | 13.4 |
| *DBH*_12_ (cm) | 314 | 17.59 (± 4.44) | 6.90 | 30.43 | 25.3 |
| *V*_12_ (m^3^) | 286 | 0.29 (± 0.15) | 0.04 | 0.76 | 52.9 |
| *HT*_15_ (m) | 283 | 24.29 (± 3.71) | 10.00 | 30.00 | 15.3 |
| *DBH*_15_ (cm) | 292 | 18.72 (± 4.36) | 7.00 | 30.70 | 23.3 |
| *V*_15_ (m^3^) | 283 | 0.35 (± 0.19) | 0.03 | 0.92 | 53.6 |
| *BD*15 (g/cm^3^) | 246 | 0.57 (± 0.03) | 0.46 | 0.62 | 4.5 |
| *NS*_0.5_ | 289 | 13.05 (± 4.82) | 1.00 | 21.00 | 36.9 |
| *HTs1*_0.5_ (m) | 289 | 2.24 (± 0.53) | 0.40 | 3.80 | 23.5 |
| *HTs2*_0.5_ (m) | 287 | 2.11 (± 0.52) | 0.60 | 4.00 | 24.8 |
| *GDs1*_0.5_ (cm) | 289 | 0.20 (± 0.05) | 0.06 | 0.38 | 26.0 |
| *GDs2*_0.5_ (cm) | 287 | 0.17 (± 0.05) | 0.04 | 0.38 | 27.5 |
| *CP*_0.5_ | 289 | 4.40 (± 1.06) | 1.00 | 6.00 | 24.0 |
| *F*_0_ | 282 | 0.23 (± 0.04) | 0.15 | 0.57 | 17.5 |
| *F*_m_ | 282 | 0.92 (± 0.17) | 0.63 | 2.29 | 18.4 |
| *F*_v_ | 282 | 0.69 (± 0.13) | 0.47 | 1.72 | 19.3 |
| *Y(II)* | 282 | 0.75 (± 0.02) | 0.70 | 0.80 | 2.6 |
| *F*_s_ | 282 | 0.35 (± 0.06) | 0.21 | 0.56 | 16.3 |
| *F*_m_' | 282 | 0.50 (± 0.09) | 0.29 | 0.83 | 17.7 |
| *F*_v_' | 282 | 0.15 (± 0.04) | 0.07 | 0.40 | 27.7 |
| *Y(II)*' | 282 | 0.29 (± 0.04) | 0.21 | 0.51 | 15.1 |
| *ETR* | 282 | 143.99 (± 26.98) | 82.17 | 262.30 | 18.7 |
| *NPQ* | 282 | 1.08 (± 1.30) | 0.09 | 20.57 | 121.1 |
| *SPADR* | 282 | 42.05 (± 3.18) | 29.84 | 57.82 | 7.6 |
| *HTs1*_1.5_ (m) | 275 | 7.77 (± 1.52) | 3.10 | 12.10 | 19.6 |
| *HTs2*_1.5_ (m) | 260 | 7.64 (± 1.50) | 2.90 | 11.20 | 19.6 |
| *DBHs1*_1.5_ (cm) | 275 | 5.54 (± 1.47) | 1.50 | 9.90 | 26.5 |
| *DBHs2*_1.5_ (cm) | 260 | 5.46 (± 1.49) | 1.50 | 8.90 | 27.2 |
| *Vs1*_1.5_ (m^3^) | 275 | 0.01 (± 0.01) | 2.65 × 10^−4^ | 0.04 | 57.2 |
| *Vs2*_1.5_ (m^3^) | 260 | 0.01 (± 0.01) | 2.40 × 10^−4^ | 0.03 | 54.3 |
| *Vs*_1.5_ (m^3^) | 275 | 0.02 (± 0.01) | 9.90 × 10^−4^ | 0.04 | 51.8 |
| *BAs*_1.5_ (^o^) | 275 | 60.54 (± 4.20) | 46.75 | 76.25 | 6.9 |
| *HTLBs*_1.5_ (m) | 275 | 3.00 (± 0.87) | 0.60 | 5.40 | 28.8 |
| *NBs*_1.5_ | 275 | 28.34 (± 6.97) | 8.00 | 51.00 | 24.6 |
| *CWs1*_1.5_ (m) | 275 | 2.94 (± 0.60) | 1.00 | 4.70 | 20.4 |
| *CWs2*_1.5_ (m) | 275 | 3.10 (± 0.70) | 1.50 | 4.70 | 21.1 |
| *CPAs*_1.5_ (m^2^) | 275 | 7.42 (± 2.62) | 1.20 | 14.10 | 35.4 |
| *CLs*_1.5_ (m) | 275 | 5.00 (± 1.11) | 1.90 | 8.60 | 22.1 |
| *HTs1*_2.5_ (m) | 269 | 10.85 (± 2.17) | 4.00 | 15.60 | 20.0 |
| *HTs2*_2.5_ (m) | 245 | 11.07 (± 2.15) | 3.70 | 15.00 | 19.4 |
| *DBHs1*_2.5_ (cm) | 270 | 7.37 (± 1.83) | 2.60 | 12.80 | 24.8 |
| *DBHs2*_2.5_ (cm) | 249 | 7.31 (± 1.89) | 2.80 | 12.00 | 25.9 |
| *Vs1*_2.5_ (m^3^) | 269 | 0.02 (± 0.01) | 1.06 × 10−3 | 0.08 | 55.4 |
| *Vs2*_2.5_ (m^3^) | 245 | 0.02 (± 0.01) | 0.97 × 10−3 | 0.06 | 53.9 |
| *Vs*_2.5_ (m^3^) | 269 | 0.04 (± 0.02) | 1.44 × 10−3 | 0.10 | 49.4 |

SD: standard deviation. Trait abbreviations can be found on the title page of this supplementary material. Significance level in ANOVA: ^***^ *P* ≤ 0.001, ^**^ *P* ≤ 0.01, ^*^ *P* ≤ 0.05, and non-significance for the rest *F* values.

**Supplementary Table 2.** Significance difference (*t*-test) between ortet and ramet sib mean performance for 53 traits investigated in a cloned *Eucalyptus* *urophylla* × *E*. *tereticornis* cross.

| **Trait** | **N** | **Mean (± SD) of ortets** | **Mean (± SD) of ramet sibs** | ***t*-test** | |
| --- | --- | --- | --- | --- | --- |
| Growth of the first-rotation | | | | | |
| *V*_1.5_ (m^3^) | 210 | 0.02 (± 0.01) | 0.02 (± 0.01) | 3.60^***^ | |
| *V*_2.5_ (m^3^) | 199 | 0.04 (± 0.02) | 0.03 (± 0.01) | 3.08^**^ | |
| *V*_4.5_ (m^3^) | 192 | 0.11 (± 0.06) | 0.09 (± 0.05) | 5.72^***^ | |
| *V*_5.5_ (m^3^) | 165 | 0.15 (± 0.08) | 0.12 (± 0.06) | 4.75^***^ | |
| *V*_6.5_ (m^3^) | 131 | 0.18 (± 0.09) | 0.16 (± 0.08) | 2.57^*^ | |
| *V*_8_ (m^3^) | 110 | 0.22 (± 0.11) | 0.19 (± 0.10) | 2.63^**^ | |
| *HT*_10_ (m) | 102 | 21.42 (± 6.03) | 22.72 (± 3.10) | −2.29^*^ | |
| *DBH*_10_ (cm) | 116 | 18.16 (± 6.46) | 17.69 (± 4.13) | 0.79 | |
| *V*_10_ (m^3^) | 102 | 0.32 (± 0.19) | 0.28 (± 0.15) | 1.87 | |
| *HT*_12_ (m) | 87 | 21.80 (± 5.98) | 22.89 (± 3.26 | −1.71 | |
| *DBH*_12_ (cm) | 114 | 19.41 (± 6.13) | 18.30 (± 4.47) | 1.86 | |
| *V*_12_ (m^3^) | 87 | 0.35 (± 0.22) | 0.30 (± 0.17) | 1.81 | |
| *HT*_15_ (m) | 76 | 23.86 (± 6.87) | 24.52 (± 3.91) | −0.82 | |
| *DBH*_15_ (cm) | 105 | 21.01 (± 6.19) | 18.99 (± 4.42) | 3.34^**^ | |
| *V*_15_ (m^3^) | 76 | 0.44 (± 0.27) | 0.35 (± 0.18) | 2.90^**^ | |
| Coppicing | | | | |  |
| *NS*_0.5_ | 105 | 17.86 (± 5.40) | 13.45 (± 4.51) | 7.57^***^ | |
| *HTs1*_0.5_ (m) | 105 | 2.38 (± 0.57) | 2.25 (± 0.42) | 2.13^*^ | |
| *HTs2*_0.5_ (m) | 105 | 2.55 (± 1.74) | 2.11 (± 0.48) | 2.47^*^ | |
| *GDs1*_0.5_ (cm) | 105 | 2.02 (± 0.57) | 0.20 (± 0.04) | 33.21^***^ | |
| *GDs2*_0.5_ (cm) | 105 | 1.75 (± 0.50) | 0.17 (± 0.04) | 32.87^***^ | |
| *CP*_0.5_ | 105 | 5.30 (± 1.22) | 4.46 (± 1.00) | 6.12^***^ | |
| Chlorophyll fluorescence and concentration | | | | |  |
| *F*_0_ | 103 | 0.22 (± 0.02) | 0.23 (± 0.04) | −1.71 | |
| *F*_m_ | 103 | 0.90 (± 0.10) | 0.93 (± 0.18) | −1.19 | |
| *F*_v_ | 103 | 0.69 (± 0.09) | 0.70 (± 0.14) | −0.99 | |
| *Y(II)* | 103 | 0.76 (± 0.02) | 0.75 (± 0.02) | 1.44 | |
| *F*_s_ | 103 | 0.37 (± 0.06) | 0.37 (± 0.06) | −0.12 | |
| *F*_m_' | 103 | 0.51 (± 0.08) | 0.52 (± 0.09) | −1.88 | |
| *F*_v_' | 103 | 0.14 (± 0.04) | 0.15 (± 0.04) | −2.52^*^ | |
| *Y(II)*' | 103 | 0.27 (± 0.05) | 0.29 (± 0.04) | −2.03^*^ | |
| *ETR* | 103 | 109.69 (± 25.34) | 141.49 (± 25.76) | −9.31^***^ | |
| *NPQ* | 103 | 0.83 (± 0.32) | 0.96 (± 0.66) | −1.86 | |
| *SPADR* | 103 | 41.15 (± 5.85) | 42.13 (± 3.13) | −1.58 | |
| Growth of the second-rotation | | | | |  |
| *HTs1*_1.5_ (m) | 97 | 6.89 (± 1.98) | 7.81 (± 1.42) | −3.83^***^ | |
| *HTs2*_1.5_ (m) | 81 | 6.91 (± 2.09) | 7.73 (± 1.49) | −2.90^**^ | |
| *DBHs1*_1.5_ (cm) | 98 | 5.20 (± 2.00) | 5.59 (± 1.39) | −1.62 | |
| *DBHs2*_1.5_ (cm) | 81 | 5.32 (± 2.05) | 5.65 (± 1.42) | −1.15 | |
| *Vs1*_1.5_ (m^3^) | 97 | 0.34 (± 0.25) | 0.01 (± 0.01) | 12.85^***^ | |
| *Vs2*_1.5_ (m^3^) | 81 | 0.33 (± 0.23) | 0.01 (± 0.01) | 12.21^***^ | |
| *Vs*_1.5_ (m^3^) | 97 | 0.61 (± 0.38) | 0.02 (± 0.01) | 15.47^***^ | |
| *BAs*_1.5_ (^o^) | 97 | 59.16 (± 8.08) | 60.17 (± 3.58) | −1.20 | |
| *HTLBs*_1.5_ (m) | 97 | 2.93 (± 0.94) | 3.10 (± 0.80) | −1.55 | |
| *NBs*_1.5_ | 97 | 28.49 (± 10.96) | 28.35 (± 5.98) | 0.12 | |
| *CWs1*_1.5_ (m) | 97 | 2.68 (± 0.85) | 2.92 (± 0.59) | −2.32^*^ | |
| *CWs2*_1.5_ (m) | 97 | 2.70 (± 0.75) | 3.08 (± 0.59) | −4.00^***^ | |
| *CPAs*_1.5_ (m^2^) | 97 | 6.04 (± 2.99) | 7.37 (± 2.52) | −3.42^***^ | |
| *CLs*_1.5_ (m) | 97 | 4.49 (± 1.56) | 4.98 (± 1.08) | −2.82^**^ | |
| *HTs1*_2.5_ (m) | 95 | 10.03 (± 2.81) | 10.80 (± 1.99) | −2.08^*^ | |
| *HTs2*_2.5_ (m) | 75 | 10.47 (± 2.85) | 11.18 (± 2.00) | −1.68 | |
| *DBHs1*_2.5_ (cm) | 95 | 7.10 (± 2.37) | 7.37 (± 1.75) | −0.84 | |
| *DBHs2*_2.5_ (cm) | 76 | 7.56 (± 2.44) | 7.56 (± 1.79) | −0.01 | |
| *Vs1*_2.5_ (m^3^) | 97 | 0.01 (± 0.01) | 0.01 (± 0.01) | −1.78 | |
| *Vs2*_2.5_ (m^3^) | 81 | 0.01 (± 0.01) | 0.01 (± 0.01) | −1.96 | |
| *Vs*_2.5_ (m^3^) | 97 | 0.02 (± 0.01) | 0.02 (± 0.01) | −2.02 | |

SD: standard deviation. Trait abbreviations can be found on the title page of this supplementary material. Significance level in *t*-test: ^***^ *P* ≤ 0.001, ^**^ *P* ≤ 0.01, ^*^ *P* ≤ 0.05, and non-significance for the rest values.

**Supplementary Table 5.** Relative genetic gain (*RG*, %; on the diagonal and in bold) of a single-trait selection and efficiency (*E*, %; above the diagonal) of a single-trait selection relative to an elder trait or another trait of the same age in a cloned *Eucalyptus urophylla* × *E*. *tereticornis* cross.

| **Selection trait** | **Selection  method** | **Number of clones selected** | **Selection differential** | ***V*_1.5_** | ***V*_2.5_** | ***V*_4.5_** | ***V*_5.5_** | ***V*_6.5_** | ***V*_8_** | ***BD*_8_** | ***CC*_8_** | ***V*_10_** | ***V*_12_** | ***V*_15_** | ***BD*_15_** | ***CP*_0.5_** | ***Vs*_1.5_** | ***Vs*_2.5_** |
| --- | --- | --- | --- | --- | --- | --- | --- | --- | --- | --- | --- | --- | --- | --- | --- | --- | --- | --- |
| *V*_1.5_ | BLUP | 60 | 0.01 | **45.1** | 92.3 | 82.0 | 80.3 | 71.4 | 70.7 | 31.4 | −0.3 | 63.5 | 58.4 | 33.0 | −250.9 | 27.3 | −10.7 | −5.1 |
| *V*_2.5_ | BLUP | 59 | 0.02 |  | **52.2** | 90.2 | 88.6 | 80.9 | 80.3 | 39.3 | 0.04 | 74.5 | 69.0 | 47.4 | −219.8 | 37.2 | −4.5 | 2.7 |
| *V*_4.5_ | BLUP | 56 | 0.05 |  |  | **72.3** | 97.0 | 88.7 | 86.8 | 53.0 | 0.1 | 83.6 | 80.6 | 59.2 | −156.6 | 43.2 | 3.8 | 7.5 |
| *V*_5.5_ | BLUP | 55 | 0.08 |  |  |  | **79.1** | 89.5 | 86.7 | 46.6 | 0.1 | 84.8 | 79.9 | 56.1 | −165.0 | 46.5 | 7.4 | 12.8 |
| *V*_6.5_ | BLUP | 49 | 0.08 |  |  |  |  | **68.1** | 95.8 | 46.2 | 0.04 | 95.2 | 90.1 | 70.4 | −185.6 | 40.1 | 0.03 | 7.1 |
| *V*_8_ | BLUP | 48 | 0.1 |  |  |  |  |  | **70.0** | 42.1 | 0.04 | 97.6 | 92.7 | 71.9 | −115.9 | 33.3 | −3.0 | 4.4 |
| *BD*_8_ | BLUP | 48 | 0.02 |  |  |  |  |  |  | **4.6** | 0.3 | 47.7 | 43.0 | 24.5 | −218.5 | 26.9 | 5.4 | 5.8 |
| *CC*_8_ | BLUP | 48 | 0.01 |  |  |  |  |  |  |  | **3.9** | −9.3 | −15.5 | −29.1 | −397.0 | −1.1 | 0.0 | 2.8 |
| *V*_10_ | BLUP | 48 | 0.13 |  |  |  |  |  |  |  |  | **63.2** | 94.4 | 71.5 | −76.8 | 34.8 | −1.9 | 10.0 |
| *V*_12_ | BLUP | 47 | 0.14 |  |  |  |  |  |  |  |  |  | **65.5** | 76.9 | −69.7 | 28.8 | −16.1 | −3.0 |
| *V*_15_ | BLUP | 44 | 0.15 |  |  |  |  |  |  |  |  |  |  | **64.1** | −120.6 | 38.2 | −11.2 | 0.7 |
| *BD*_15_ | BLUP | 44 | 0.01 |  |  |  |  |  |  |  |  |  |  |  | **3.4** | −3.8 | −13.6 | −6.5 |
| *CP*_0.5_ | BLUP | 43 | 0.52 |  |  |  |  |  |  |  |  |  |  |  |  | **16.1** | 8.3 | 21.2 |
| *Vs*_1.5_ | BLUP | 41 | 0.003 |  |  |  |  |  |  |  |  |  |  |  |  |  | **34.5** | 88.5 |
| *Vs*_2.5_ | BLUP | 41 | 0.008 |  |  |  |  |  |  |  |  |  |  |  |  |  |  | **38.8** |

BLUP: best linear unbiased prediction. Trait abbreviations can be found on the title page of this supplementary material.
